# Rare intercostal herniation of abdominal organs in COPD patient managed non-operatively

**DOI:** 10.1093/jscr/rjae217

**Published:** 2024-07-11

**Authors:** Dalya M Abdelmaged, Thomas Litton

**Affiliations:** Department of General Surgery, HCA Healthcare, Charleston, SC 29406, United States; Department of General Surgery, HCA Healthcare, Charleston, SC 29406, United States

**Keywords:** intercostal hernia, COPD, intrathoracic herniation

## Abstract

Nontraumatic intercostal and intrathoracic herniation of intra-abdominal organs is rare and has been sparsely reported in the literature. They are defined as protrusion of intra-abdominal contents through defects in the chest wall. The cases reported in the literature mostly involved herniation of intra-abdominal contents and the lungs through the defect. In this case report, we describe a case of intra-abdominal contents herniation through an intercostal defect with subsequent improvement in patient’s respiratory status related to the hernia.

## Introduction

Intercostal herniation of intra-abdominal contents is very rare and pose diagnostic and management challenges. They are protrusion of abdominal organs through a defect in the chest wall [1]. They are categorized into acquired (traumatic and nontraumatic) and congenital, or classified into thoracic, abdominal, or trans-diaphragmatic subtypes. This hernia is usually addressed surgically, especially in young patients, and in cases of organ perforation or strangulation. Non-operative management is reserved for poor surgical candidates who are asymptomatic or have mild symptoms. In this report, we explore the diagnostic and treatment challenges of this hernia and present a patient with chronic obstructive pulmonary disease (COPD) whose respiratory status improved following intercostal herniation and expose a unique perspective to evaluating these defects in an individualized approach.

## Case report

A 76-year-old female with a 50 pack-year smoking history and COPD (for 12 years) on supplemental oxygen and tiotropium/olodaterol, presented to the emergency department with increasing abdominal distention and obstipation for 7 days. The patient also noted that the caliber of her stools had been getting smaller but remained non-bloody and without melena. Patient’s last colonoscopy was 9 years prior to presentation with benign polyps.

Three days before admission, the patient was seen in an outside facility with increasing dyspnea and diagnosed with pneumonia. The patient reported that while at that facility she was transferred from one stretcher to another and was dropped into her left flank. The patient then noticed pain and extensive bruising over the left flank that continued to expand in size, but denied pain in the abdomen.

**Figure 1:**
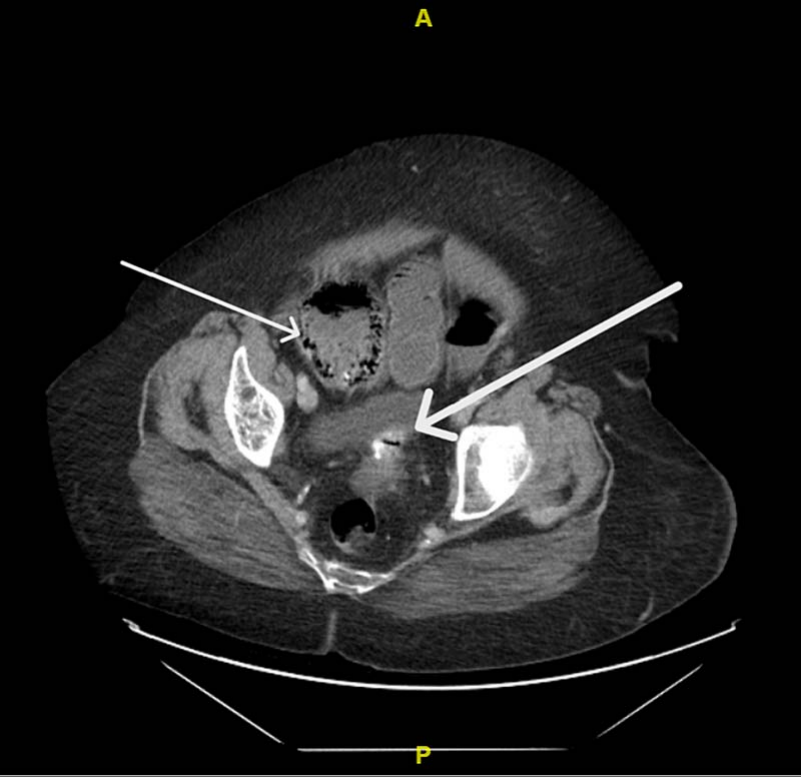
Pneumatosis intestinalis of the right colon (thin arrow) and sigmoid mass (thick arrow).

**Figure 2:**
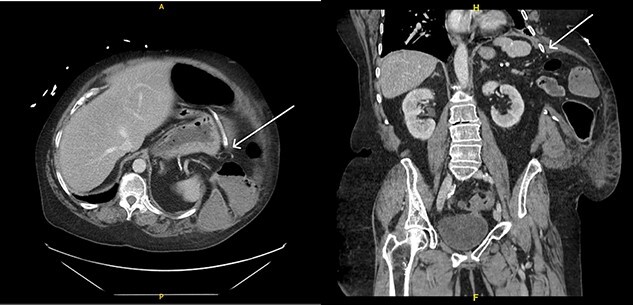
Left intercostal hernia containing loops of large bowel, axial view (left), coronal view (right).

Upon evaluation, the patient was found to have white blood cells 20.1 (4–10.9), with unremarkable vitals and no signs of peritonitis on examination. Computerized tomography (CT) abdomen/pelvis revealed a large bowel obstruction with pneumatosis intestinalis of the right colon secondary to a sigmoid mass (Fig. 1). Upon surgical review of the images, the patient’s CT also showed a left-sided intercostal hernia containing loops of large bowel that was not initially reported by the radiologist (Fig. 2). The mechanism of this was unclear; however, it has been hypothesized that it could be a result of a combination of severe cough related to patient’s recent pneumonia and underlying COPD, or trauma to the left chest wall allegedly sustained at the previous facility.

**Figure 3:**
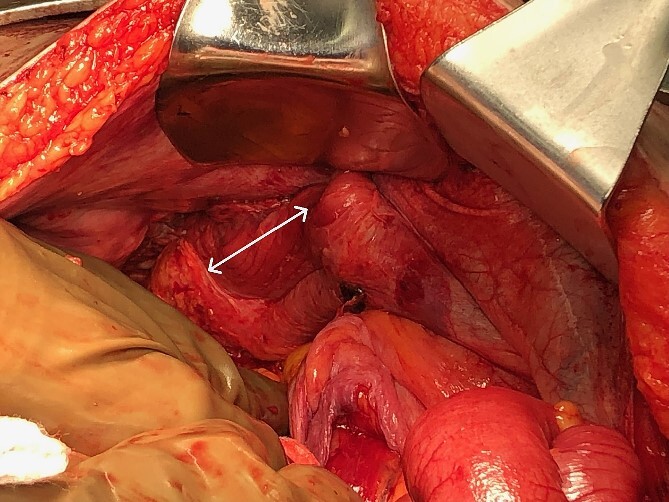
Part of the thoracic defect as seen intraoperatively (arrows).

The patient was taken urgently to the operating room and underwent exploratory laparotomy with total abdominal colectomy and end ileostomy. Operative findings confirmed a large lateral intercostal defect between the 9th and 10th ribs (Fig. 3), as well as ischemic changes of the ascending and transverse colon due to an obstructing mass in the mid-sigmoid, which subsequently proved to be a benign diverticular stricture. The patient progressed well postoperatively and had an uneventful hospital course. Post-discharge, the patient’s transthoracic hernia was found to be unchanged in size by repeat CT, and it had actually improved her lung function likely due to improvement in diaphragmatic excursion. The patient did not have a pre-operative baseline pulmonary function test; however, postoperatively, her FEV_1_/FVC was 70%, and clinically the patient showed noticeable improvements compared to preoperative including cessation of supplemental oxygen therapy and improved functional status. With patient in standing position, compressing the hernia made her noticeably short of breath. We discussed surgical repair, but advised against it given her comorbidities, the asymptomatic nature of this hernia, and the noticeably improved respiratory and overall functional status as mentioned above. The patient agreed to the plan and subsequently underwent ileostomy takedown with ileorectal pouch anastomosis 9 months later. Postoperatively, the patient was doing well, ambulating without assistance and without supplemental oxygen.

## Discussion

Intercostal hernias of intra-abdominal contents are very rare occurrences. The negative intrathoracic pressure leads to herniation of abdominal content into the chest cavity [2]. They are linked to sudden increase in thoracoabdominal pressure such as violent coughing spells and heavy lifting [1]. In most published case reports, coughing was the most common risk factor [1]. The intercostal defects are mostly located under the ninth rib without significant differences as to the side of herniation [3]. The main symptoms are chest swelling, discomfort, and pain [3]. Examination usually reveals a bulge or bruise over the side of the chest [1]. In addition, the area of the defect can behave like a flail segment leading to bulging of lung through the defect leading to ineffective expiration [2]. Ideally, symptomatic patients are managed by surgical repair if surgery can be tolerated [2]. In a systemic review, most patients underwent surgical repair, mostly with open technique, while fewer patients underwent laparoscopic repair, with or without prosthesis [3]. Recurrence occurred in 28.6% of patients during a mean follow-up of less than a year [3].

Repair of this hernia in our case was deferred because there was no evidence of intra-abdominal organ injury, patient’s respiratory and functional status had significantly improved, and a close follow-up was implemented. A key learning point from this case report is to manage these hernias in an individualized patient-centered approach. Another key point is to maintain a high threshold for suspicion of intercostal and thoracic herniation especially in patients with COPD and other lung conditions and those with risk factors causing weak abdominal and thoracic muscles. For example, a report described a case of extensive transdiaphragmatic intercostal hernia involving the lung and intrabdominal organs induced by coughing in a patient with asthma on long-term steroids [4]. It is also essential to emphasize the importance of personally examining CT scans images in addition to reviewing the radiology report, as in this case the presence of the intercostal hernia was not reported by two radiologists on two consecutive CT exams. The rare incidence of these hernias makes them a diagnostic and management challenge, and delay in management can result in poor outcome. Herniation of any organ or structure with strangulation is a surgical emergency. However, there is a place for non-operative management for patients who present without the aforementioned findings especially those who are poor surgical candidates. The outcome of conservative management in young fit patients remains unclear, which led to some surgeons recommending surgical management of those patients.
